# Reirradiation of Locally Recurrent Prostate Cancer with Cyberknife^®^ System or Volumetric Modulated Arc Therapy (VMAT) and IGRT-Clarity^®^: Outcomes, Toxicities and Dosimetric Evaluation

**DOI:** 10.3390/cancers14133187

**Published:** 2022-06-29

**Authors:** Rossella Di Franco, Valentina Borzillo, Esmeralda Scipilliti, Gianluca Ametrano, Marcello Serra, Cecilia Arrichiello, Federica Savino, Fortuna De Martino, Valentina D’Alesio, Fabrizio Cammarota, Anna Crispo, Sandro Pignata, Sabrina Rossetti, Giuseppe Quarto, Paolo Muto

**Affiliations:** 1 Department of Radiation Oncology, Istituto Nazionale Tumori—IRCCS—Fondazione G. Pascale, 80131 Napoli, Italy; v.borzillo@istitutotumori.na.it (V.B.); esmeralda.scipilliti@istitutotumori.na.it (E.S.); gianluca.ametrano@istitutotumori.na.it (G.A.); marcello.serra@istitutotumori.na.it (M.S.); c.arrichiello@istitutotumori.na.it (C.A.); valentina.dalesio@istitutotumori.na.it (V.D.); p.muto@istitutotumori.na.it (P.M.); 2 LB Business Services RSL, 00124 Rome, Italy; federica.savino@gmail.com (F.S.); fabrizio.cammarota@gmail.com (F.C.); 3 Dipartimento di Scienze Biomediche Avanzate, Università degli Studi di Napoli Federico II, 80131 Napoli, Italy; fortuna_demartino@outlook.it; 4 Epidemiology and Biostatistics Unit, Istituto Nazionale Tumori—IRCCS—Fondazione G. Pascale, 80131 Napoli, Italy; a.crispo@istitutotumori.na.it (A.C.); s.rossetti@istitutotumori.na.it (S.R.); 5 Departmental Unit of Clinical and Experimental Uro-Andrologic Oncology, Istituto Nazionale Tumori—IRCCS—Fondazione G. Pascale, 80131 Napoli, Italy; s.pignata@istitutotumori.na.it; 6 Department of Uro-Gynecological, Istituto Nazionale Tumori—IRCCS—Fondazione G. Pascale, 80131 Napoli, Italy; g.quarto@istitutotumori.na.it

**Keywords:** prostate cancer, radiotherapy, re-treatment, Cyberknife^®^ system, VMAT, IGRT-Clarity^®^

## Abstract

**Simple Summary:**

From April 2017 to December 2020, 29 patients with prostate cancer recurrence were selected. They received Cyberknife^®^ (CK) treatment (17 pts) or alternatively VMAT (Volumetric Modulated Arc Technique) therapy by IGRT (Image-Guided Radiation Therapy)/Clarity^®^ (12 pts). Urinary (GU), rectal (GI) toxicities, and biochemical control were investigated. Further, the two techniques were dosimetrically compared by rival plans. The VMAT-IGRT Clarity^®^ treatments were replanned applying a template developed for prostate VMAT-SBRT in FFF mode, keeping the same dose/fractionation scheduled for the CK Group (30 Gy in 5 fx at 80% isodose). In the CK group, 23% of patients experienced grade 2 acute GU, while 6% showed grade 2 acute GI. In the VMAT-Clarity^®^ group, 17% of patients showed acute GU toxicity, while for 8% grade 2 late toxicity was recorded. The dosimetric analysis shows that VMAT-FFF allows to deliver a biological equivalent dose to CK, with the advantage of reducing the likelihood of toxicities arising.

**Abstract:**

The management of prostate cancer recurrence following external beam radiotherapy is not defined yet. Stereotaxic body reirradiation therapy showed encouraging results for local and biochemical control. From April 2017 to December 2020, 29 patients with prostate cancer recurrence were collected, joining the retrospective studies CyPro (prot. 46/19 OSS) and CLARO (Prot. 19/20 OSS) trials. Patients received Cyberknife^®^ treatment (17 pts) or alternatively VMAT (Volumetric Modulated Arc Technique) therapy by IGRT (Image-Guided Radiation Therapy)/Clarity^®^ (12 pts). By comparing the reirradiation of two groups, urinary (GU), rectal (GI) toxicities, and biochemical control were investigated. Further, the two techniques were dosimetrically compared by rival plans. The VMAT-IGRT Clarity^®^ treatments were replanned with an optimized template developed for prostate VMAT-SBRT in FFF mode keeping the same dose and fractionation scheduled for Cyberknife Group (30 Gy in 5 fx, at 80% isodose). In the CK group, 23% of patients experienced grade 2 acute GU, while 6% grade 2 acute GI. In the VMAT-Clarity^®^ group, acute GU toxicity was recorded in 17%, while for 8% grade 2 late toxicity was recorded. The dosimetric analysis shows that the VMAT-FFF allows to deliver a biological equivalent dose to CK, with the advantage of reducing the likelihood of toxicities arising.

## 1. Introduction

No standardization for the optimal management of locally recurrent prostate cancer after a prior radiotherapy is actually provided. Among the therapeutic options, a radical salvage prostatectomy has been employed in selected cases, but with possible local complications; cryosurgery or high-intensity focused ultrasound (US), may be considered, but their use is controversial for adverse effects, such as fistulas or rectal damage [1].

Systemic treatment such as Androgen Deprivation Therapy (ADT) remains the lifelong therapeutic choice for many patients. This therapy has a major negative impact on quality of life and can induce a more aggressive CRPC phenotype [2,3,4,5].

Salvage reirradiation for recurrent prostate cancer after primary EBRT has been limited due to toxicity to adjacent organs, particularly for the rectum and bladder [6,7,8]. 

A dose escalation on tumors with limited toxicities on adjacent organ at risks (OARs) has been allowed by the use of advanced technological modalities and techniques, such as VMAT, SBRT, and IGRT [9,10].

The use of prostate magnetic resonance imaging (MRI) allows to identify the localization of local recurrence with higher precision. SBRT represents nowadays an interesting locoregional treatment option for limited sites of recurrence [11].

We present our preliminary experience of reirradiation using stereotactic body radiotherapy, for local recurrence of prostate cancer, focusing on early rectal and bladder toxicities, as well as the initial patterns of PSA response. 

The aim of our study was to compare the prostate cancer reirradiation performed by stereotactic ablative radiotherapy with Cyberknife^®^ vs. VMAT/IGRT-Clarity^®^, relating urinary and rectal toxicities and biochemical control. Further, rival plans achieved by the two techniques were dosimetrically compared.

## 2. Materials and Methods

### 2.1. Patient Selection

Joining the retrospective studies CyPro (prot. 46/19 OSS) and CLARO (Prot. 19/20 OSS) Trials, from April 2017 to December 2020, we collected 29 consecutive patients (pts) with prostate cancer recurrence. The following eligibility criteria were employed: a biochemical failure plus four years after definitive radiotherapy, as Phoenix definition (except for pts who had macroscopic recurrence in the prostatic bed); diagnosis of local recurrence with Coline-PSMA, PET/TC, and mp-MRI (multiparametric-Magnetic Resonance Imaging) and PSA (Prostate Specific Antigen) lower than 15 ng/mL at the time of recurrence (within 2 months prior to enrollment). Eligible pts required an ECOG of 0–1, with a life expectancy of 10 years or more. Patients with grade 3 or more toxicity from previous radiotherapy were excluded. Patients enrolled from 2017 to 2018 were treated with Cyberknife^®^. From 2019, with the innovation of VMAT by IGRT/Clarity^®^, pts enrolled could take advantage of a stereotactic treatment. Therefore, the two rival approaches were used alternatively, preferring VMAT in patients who were unable to have fiducial markers, or in patients poorly compliant for long-term treatments. While, treatment with Cyberknife^®^ was preferred in those patients whit low quality images by prostate ultrasound. Among the pts enrolled for SBRT reirradiation, 17 were treated with the Cyberknife^®^ System and 12 went through VMAT/IGRT-Clarity^®^.

### 2.2. First Irradiation

Before the first treatment, 5 patients belonged to the low-risk group, 8 to the intermediate-risk group, 14 to the high-risk group and for two patients it was unknown.

All patients were treated with radical intention; two patients disclosed bone oligo-metastatics. The Gleason Score and T stage of patients before the first treatment are shown in Table 1. 

Overall, 25 patients underwent EBRT, 3 SBRT, and 1 brachytherapy (BT). In the EBRT subgroup, the median total dose (TD) was 74 Gy (64–84 Gy). The fraction dose selected was 1.8–2 Gy. The SBRT patients received a TD of 35 Gy/5 fx. The BT patient received a TD of 38 Gy/4 fx (high-dose rate (HDR) by ^192^Ir source). Detailed data concerning prior RT are presented in Table 1. Eight of them were irradiated on the pelvis with a median dose of 50.4 Gy (range 48.6–51).

The PSA nadir after the first definitive radiotherapy ranged from 0 to 3.1 ng/mL (mean 0.55 ng/mL, median 0.22 ng/mL). 

### 2.3. Salvage SBRT: Planning and Delivery

In total, 27 patients were reirradiated with an equivalent regimen, a total dose of 30 Gy in 5 fractions (6 Gy/fx) was delivered. One patient was reirradiated (on intraprostatic lesion) with a total dose of 35 Gy in 5 fractions (7 Gy/fx), another one was reirradiated (on intraprostatic lesion) with a total dose of 30 Gy in 3 fractions (10 Gy/fx).

A subgroup of 17 patients was treated with the Cyberknife^®^ System. For these patients, four gold fiducial markers were implanted transrectally into the prostate, guided by ultrasound images to verify and adjust the treatment to the potential prostate movement. A no-contrast simul-CT (Aquilon CT system; slice: 1 mm) was acquired 10 days after fiducial placement in supine position with a personalized immobilization system (vac-lok). The treatment plan was developed by the Precision^®^ inverse treatment planning system (Accuray Inc., Sunnyvale, CA, USA), the prescription dose was normalized to the 80% isodose line. 

The remaining subgroup of 12 patients was treated with VMAT and 3D-US reference scan (Clarity^®^ System, Elekta, Stockholm, Sweden), which requires a CT simulation equipped with Clarity^®^ ultrasound. The Clarity^®^ System was used in the simulation and treatment phase, providing no additional dose to the patients [12,13]. 

The Clarity^®^ System was composed of two mobile units, one in the CT room and the second in the treatment room, connected to a workstation/server. The workstation was used for target delineation and prostate monitoring. A ceiling-mounted infrared (IR) camera recognizes the US probe by detecting IR reflectors. A simul-CT and a 3D-US reference scan were acquired for each treatment plan. Both scans were acquired using the supine patient position, with kneefix, foot support, and the transperineal ultrasound (TPUS) probe positioned. Treatments were scheduled at VERSA HD Linac (Linear Accelerators—Elekta, Stockholm, Sweden) and Raystation (RaySearch Laboratories, Stockholm, Sweden) was selected as TPS (Treatment Planning System) to realize VMAT plans. The dose distribution was normalized as 95% of the prescription dose, to cover the 95% of the PTV (Planning Target Volume). Thereafter, the CT image, contours, and treatment plan were sent to the Clarity^®^ planning workstation and recorded in the US reference scan. The transperineal imaging system (Clarity^®^, Elekta, Stockholm, Sweden) enables the acquisition of volumetric ultrasound images for pre-treatment target localization (daily IGRT) or for intra-fractional monitoring of the prostate. Before treatment delivery start, a 3D guidance ultrasound image was acquired for each session. The prostate was manually identified based on the predefined reference ultrasound images and the image guidance volume. The Clarity^®^ System calculates a 3D vector of displacement between the treatment isocenter and the prostate center, reflected in the reference ultrasound image. After applying the 3D shift vector, the system was able to monitor all subsequent 3D displacements of the prostate with respect to the reference image captured as a guide image. 

For both the techniques, target volumes delineation was performed using a simul-CT scan fused with MRI. The Gross Target Volume (GTV) was defined as whole prostate gland or prostatic bed lesion or intraprostatic lesion; Clinical Target Volume (CTV) was equal to the GTV. The PTV of the prostate gland was defined as expanded CTV of 3 posteriorly and 5 mm in all directions. The PTV of the intraprostatic lesion was defined as CTV with a 3 mm expansion in all directions. The rectum, bladder, penile bulb, femoral heads, bowel, testicles, and neurovascular bundle were contoured as organs at risk (OARs). The dose constraints for OARs are summarized in Table 2.

### 2.4. Bladder and Rectal Preparations

To increase the daily reproducibility of the configuration in terms of bladder and rectum filling, all patients were trained to drink 500 mL of water 30 min before the CT scan imaging as well as before each treatment fraction, and to perform an enema from 2 days. We prescribed Simethicone 40 mg, twice a day to all patients. Urinary and rectal toxicities were evaluated at the radiotherapy starting point, intra-treatment, and therefore every three months. Acute and late urinary and rectal toxicities according to EORTC/RTOG scale were evaluated, and the percentages of toxicity are shown in histograms.

### 2.5. Dosimetric Evaluation

Aiming to perform a dosimetric study, the VMAT-IGRT Clarity^®^ treatments were replanned with an optimized template developed for prostate VMAT-SBRT in FFF mode, keeping the same dose prescription and fractionation scheduled for the CK Group (30 Gy/5 fx, prescription isodose 80%). To increase the agreement between VMAT-SBRT in FFF and CK plans, D_max_ of VMAT plans was rescaled at 97.5% of the dose (corresponding to the average value of the PTV coverage percentage of the CK plans), to correct for our specific plan template.

The OARs doses between the two plan strategies were compared to evaluate if this new approach could be more favorable in dosimetric terms and therefore radiobiologically equivalent. The VMAT plans were computed for an VERSA-HD Linac (Elekta, Crowley, UK) equipped with 6 MV photon beam in FFF mode and MLC with a 5 mm leaf. The FFF plans were not delivered on pts, but only calculated for study purposes.

### 2.6. Literature Review

We reviewed the literature on the techniques used, on doses, targets, toxicities, and local control/failure to compare our data with other experiences. “Prostate cancer recurrence” AND “stereotactic radiotherapy” AND “retreatment” was used as string of search from 2010 to 2022. The search was supplemented with hand searches of reference lists for all available review articles and primary studies, to identify other studies not found in the computer search. The present systematic review was performed with recommendations of the Preferred Reporting Items for Systematic Reviews and Meta-Analyses (PRISMA) [14].

The included studies were prospective or retrospective, analyzing more than 5 reirradiated patients. Abstracts, reviews, case reports, no-English language articles, and animal studies were excluded. For each study, the following elements were assessed: age of patients at first diagnosis, first radiotherapy technique used, median time to relapse, median follow-up, median pre-SBRT PSA, type of SBRT, total dose and fractionation used, gastrointestinal and genitourinary toxicity, local and metastatic control/failure, and constraints used.

### 2.7. Trial Approval

The CyPro Trial (CyberKnife^®^ Prostate cancer), prot. 46/19 OSS, was approved by the Ethics Committee on 15 January 2020 (D. n. 105 of 12 February 2020), while the CLARO Trial (Clarity^®^ System in Radiation Oncology), Prot. 19/20, was approved by the Ethics Committee on 6 May 2020 (D. n. 497 of 20 May 2020). 

## 3. Results

Table 3 shows the main characteristics of patient candidates for retreatment, including the imaging used for restaging, the RT techniques, and total dose/fraction used.

### 3.1. Oncologic Outcome

The median follow-up was 27 months (range, 6–60). Regarding the median pre-SBRT PSA level of 1.9 ng/mL, at 3 months, 26/29 patients (90%) showed a biochemical response to treatment with a median PSA decline of 23% (from 1.8 to 0.42 ng/mL); 3 patients (10%) experienced early PSA progression with a median PSA elevation from 7.76 to 17 ng/mL. At 6 months, 25/29 patients (86%) showed a biochemical response to treatment with a median PSA decline of 20% (from 1.8 to 0.36 ng/mL); 4 patients (14%) experienced early PSA progression at 6 months, median PSA elevation from 7.43 to 21.5 ng/mL. At 1 year, 21/27 patients (78%) experienced a decreased median PSA from to 1.79 to 0.2 ng/mL and at 2 years 15/18 patients (83%) (2 pts returned to biochemical control after ADT initiation) from 1.9 to 0.22 ng/mL. 

**Figure 1:**
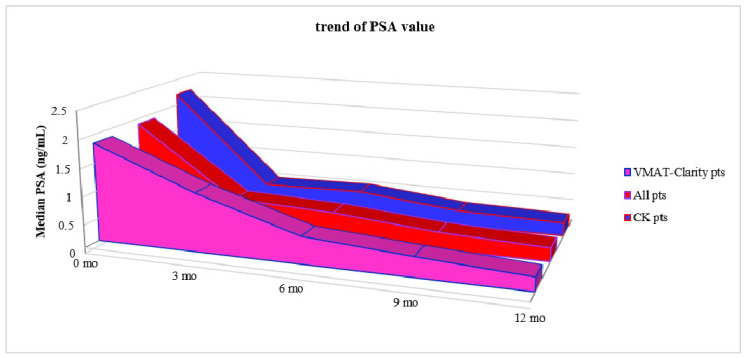
Median PSA in all patients, CK and Clarity^®^ groups.

Although more than half of the patients had a follow-up of at least 24 months, we present below (Figure 1) only the data relating to 1 year, because not all the patients of the second group reached a follow-up of 24 months, in order to carry out a balanced comparison and guarantee reliable data analysis.

Figure 1 shows the trend up to 1 year of the median PSA value of all patients compared with the group of patients treated with CK and patients treated with VMAT-Clarity^®^. No statistical correlation was found between the response to treatment-related and pretreatment variables.

We calculated the percentage of freedom from Local Recurrence, Distant Recurrence, Distant Metastases, and Biochemical Relapse over 1 year (Table 4). 

The 1-year actuarial local, distant relapse-free, androgen deprivation therapy, and biochemical relapse rates of patients are shown in Table 4. 

### 3.2. Toxicities

**Figure 2:**
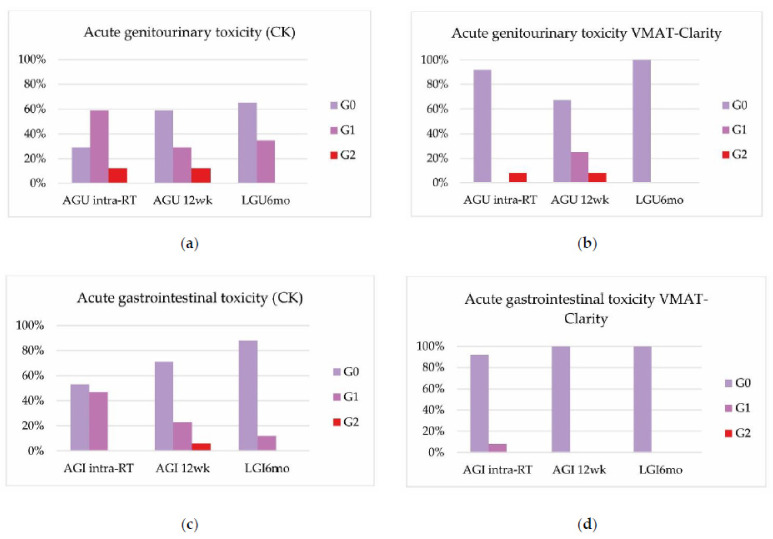
Acute genitourinary toxicity (AGU) in Cyberknife^®^ (**a**) and VMAT-Clarity^®^ (**b**) patient groups. Acute gastrointestinal toxicity (AGI) in Cyberknife^®^ (**c**) and VMAT-Clarity^®^ (**d**) patient groups.

**Figure 3:**
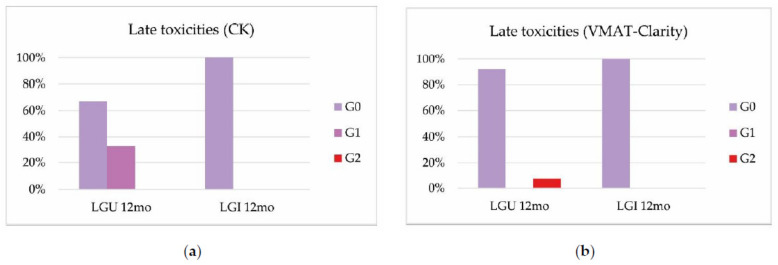
Late genitourinary (LGU) and gastrointestinal (LGI) toxicity in Cyberknife^®^ group (**a**) and VMAT-Clarity^®^ group (**b**).

Treatment-related toxicities are summarized in Figure 2 and Figure 3. 

Acute genitourinary toxicity was mild, showing in the subgroup treated with the Cyberknife^®^ system an incidence of 12% of acute grade 2 post-SBRT intra-RT and at three months. No grade 3 or higher acute toxicities were detected (Figure 2a). In the subgroup treated with VMAT-Clarity^®^, we found 8% of acute grade 2 toxicity Intra-RT and at three months (Figure 2b).

Acute gastrointestinal toxicity in the Cyberknife^®^ system group resulted in 6% G2 at 3 months (Figure 2c). In the group treated with VMAT-Clarity^®^, no toxicity greater than G1 was recorded (Figure 2d). 

At 1 year, genitourinary and gastrointestinal toxicities grade 2 in the Cyberknife^®^ system group were not recorded (Figure 3a), while 8% grade G2 genitourinary toxicity was recorded in the VMAT-Clarity^®^ group. (Figure 3b)

No gastrointestinal toxicity greater than grade 1 was recorded in either group (Figure 3a,b).

### 3.3. Dosimetric Data

By comparing the volumes (GTV and PTV) of the two groups, no significant differences were found in gland volume for patients undergoing prostatic radiotherapy. On the other side, in patients treated in sites of macroscopic recurrence after surgery or in patients treated on intraprostatic lesions, we need to take into account that the volumes were different and not easily comparable (Table 5).

Regarding the dosimetric evaluations on the organs at risk, the table shows, as predicted, that the D_max_ at PTV in CK are higher than in VMAT plans, according to the different isodose prescription. In the CK plans, the median values for V_15 Gy_, V_21 Gy_, V_24 Gy_, and V_27 Gy_ of the rectum, and V_15 Gy_ of the bladder were lower than in VMAT plans. VMAT Clarity^®^ treatments had a lower median value for V_30 Gy_ bladder and V_30 Gy_ rectum. The median of D_max_ to the penile bulb was instead greater in VMAT treatments, due to the proximity of the organ to the target resulting from the positioning of the transperineal probe. 

Aiming to obtain a dosimetric comparison, we re-planned the VMAT plans with the FFF technique to simulate the dose drop obtained with the Cyberknife^®^. For this reason, a plan with a prescription of 30 Gy in 5 fractions with an isodose of 80% was made.

The data reported in Table 5 show, for the simulated stereotaxic treatment in comparison with the Cyberknife^®^ System, an increase in D_max_ at PTV, while the median values of V_15 Gy_ of the bladder, and V_15 Gy_, V_21 Gy_, V_24 Gy_, and V_27 Gy_ of the rectum were reduced. The dose to the penile bulb was slightly increased. The dosimetric data of the prostatic beds and intraprostatic lesions are not equally comparable for too different volumes.

### 3.4. Literature Search

**Figure 4:**
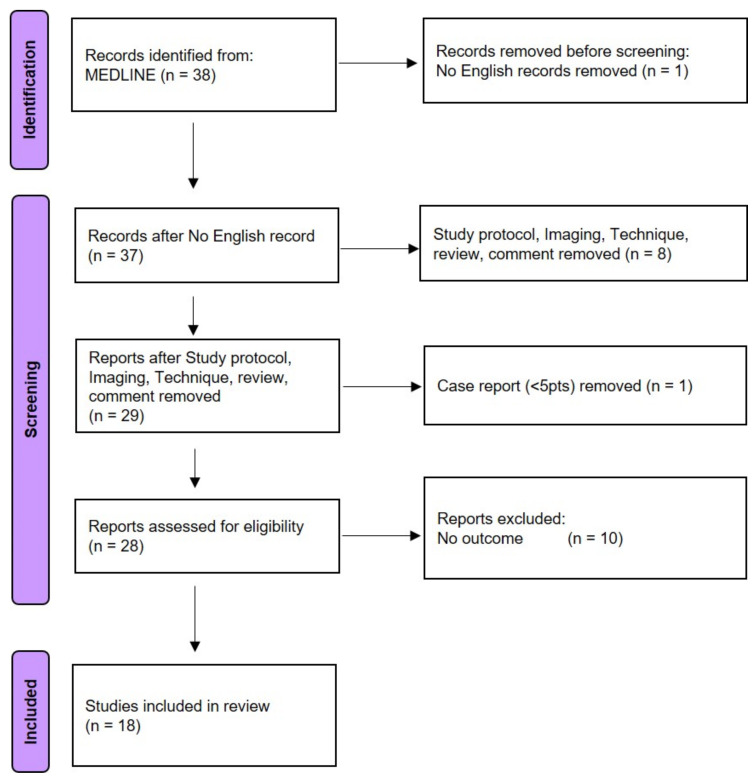
Preferred Reporting Items for Systematic Reviews and Meta-Analyses (PRISMA) used for literature review.

The search of the literature yielded 38 citations. Of these, 18 studies, published between 2015 and 2021, met the inclusion criteria (Figure 4).

The main features of the studies included in this systematic review are shown in Table 6. 

Only two studies were prospective trials [10,16]. The analyzed population of each study varied greatly, ranging from 6 [30] to 64 [27] patients.

Overall, the 18 studies included 511 patients who were reirradiated on the prostate gland, prostatic bed, or intraprostatic lesion, for PC recurrences. The median values of follow-up from retreatment in studies specifically examining reirradiated patients ranged from 9.5 to 77.6 months (median 22.5 months).

In total, 10 studies reported data regarding retreatment with CK [9,10,16,17,18,19,22,23,24,30,31], 3 with VMAT [21,26,29], 1 with CK or BT [20], 1 IMRT/VMAT [25], 1 Tomotherapy [15], 1 CK or VMAT [28], and 1 IMRT or CK [27]. The first radiation treatment was delivered with only EBRT in 10 studies [9,15,18,19,24,26,28,30,31] as exclusive or adjuvant treatment, 9 studies reported data on treatment with EBRT and/or BT [10,17,20,21,22,23,25,27,29], only 1 study reported 1 treatment performed with SBRT [19], 1 study reported treatment with EBRT including 3DCRT, IMRT, and PT [16].

The median values of time elapsed since previous irradiation ranged from 6.5 [31] to 126 months (median 88 months) [24]. Reirradiation prescription doses ranged from 15–18 Gy/3 fx [15,21] to 38 Gy/6 fx [25]. ADT was given with reirradiation in 132 patients (29%), collecting the 16 studies reporting this finding [10,16,17,18,19,20,21,22,23,24,25,26,27,29,30,31].

In terms of efficacy, the rates of BRFS (Biochemical Relapse Free Survival) and DMFS (Distant Relapse Free Survival) were analyzed at 2 years by 12 studies [9,10,16,17,18,21,22,25,26,27,28,29,31], (Table 7). Toxicities were assessed by the CTCAE version 3.0 scale [10,16], or 4 [9,18,19,20,22,24,25,26,28,29,31], or by the RTOG/EORTC scale [17,21,23,25,27,28,30].

Regarding acute genitourinary toxicities: grade ≥ 3 were observed in 8 studies [9,10,17,22,23,26,27,29] with a median percentage of 3.35% (1.5–7.9%); grade 2 in 14 studies [9,16,17,19,20,21,22,23,24,25,26,27,29,31] with a median percentage of 8.55% (2–40%). 

Regarding late genitourinary toxicity: grade 3 was observed in 8 studies [10,16,17,18,23,26,27,29] with a median percentage of 3% (1.5–12.5%); grade 2 in 12 studies [10,16,17,18,20,21,23,25,27,28,29,31] with median percentage of 9% (3–30%).

Acute gastrointestinal (GI) toxicity: grade 3 occurred only in two studies [25,28] with a mean percentage of 6.5% (4–9%); grade 2 in 6 studies [20,21,22,24,27,31] with a median percentage of 7% (2–11.1%). Late gastrointestinal toxicity: grade 3 was observed in 1 study [19] with a percentage of 2% (3.2–12.5%); G2 in 5 studies [18,20,23,27,29] with a median percentage of 4% (1.5–5.6%). 

Finally, we included in the last line of the Table 5 and Table 6 the data related to our experience to make a comparison on the outcomes, on tolerance and toxicities. Regarding our constraints, some of them were found from other authors, such as D_30%_ for the bladder or D_30%_ and D_60%_ of the rectum [21]. The constraints of the rectum V_30_, V_27_, and V_24_ can be found in the work of Lewin [29], while the V_29 Gy_ of the penile bulb was found in the work of Detti [24], and the V_24 Gy_ in D’Agostino [26].

## 4. Discussion

The management of prostate cancer recurrence after external beam radiotherapy is not defined yet. Androgen deprivation is the most common therapeutic option in salvage after curative radiotherapy, although its long-term side effects may affect patients’ quality of life. Local therapy could reduce the side effects of systemic therapies in patients with recurrent prostate cancer. 

The retreatment could be delivered in different ways, including IMRT and/or SBRT, which can be performed with different delivery systems including LINAC. SBRT allows to reduce the safety margins of the PTV, minimizing the exposure of normal irradiated tissues and to reduce treatment time.

Among the local treatments, stereotaxic body reirradiation therapy with CyberKnife^®^, for patients with local recurrence of prostate cancer after EBRT, has shown encouraging results in terms of local and biochemical control. There are numerous experiences of stereotaxic retreatment also with volumetric techniques. There is no experience in the literature regarding the use of the VMAT technique with IGRT-Clarity^®^.

In this retrospective study, we evaluated the toxicity and feasibility of a prostatic reirradiation after failure of definitive radiotherapy. Two modalities were investigated: SBRT using CyberKnife^®^ and treatment with VMAT with IGRT-Clarity^®^.

Our data shows results that agree with the literature. The dose employed in our experience for the re-treatment of the prostate gland was used in most of the works, although in the literature there are experiences using higher doses such as 32.5 Gy/5 fx [29] with the Cyberknife^®^ system, or 36 Gy/6 fx or 38 Gy/6 fx [25] with VMAT/IMRT. 

For treatments, we used the same dose in the Cyberknife^®^ and in VMAT-Clarity^®^ modality, although in the first approach the prescription was made at an isodose of 80%. This could explain the lower acute toxicity recorded in the VMAT-Clarity^®^ treatment, the same trend that we can see in the literature data reported in Table 6.

In our series, the biochemical response rate at 1 year was 81%, according to the average found by the other authors (Table 5). 

This treatment is well-tolerated in the Cyberknife^®^ group, where only 23% of patients experienced grade 2 acute genitourinary and 6% experienced grade 2 acute gastrointestinal. In the VMAT-Clarity^®^ group, only 17% acute genitourinary toxicity was recorded. 

Regarding the late toxicity, we recorded a percentage of 33% of genitourinary toxicity grade 1 in the Cyberknife^®^ group and of 8% grade 2 in the VMAT-Clarity^®^ group, while we did not register any gastrointestinal toxicity higher than G1 in both groups. We did not have any grade 3 toxicities; this result can be explained by use of high-precision radiotherapy in all patients. Indeed, the technique allowing for maximum normal-tissue sparing should be employed when reirradiation is indicated. The main limitations of this study are the small number of patients included, the retrospective nature of the study, and the short duration of follow-up especially in the group treated with VMAT-Clarity^®^.

Despite these limitations, the dosimetric analysis were performed opening a new horizon for future studies, since the use of the VMAT-FFF will not only allow the patients to deliver a biologically equivalent dose to that delivered with the Cyberknife^®^, but also to reduce the toxicities, giving the fundamentals to develop the dose [32,33].

## 5. Conclusions

This study shows that SBRT may be a promising treatment option for isolated macroscopic local recurrence after RP and EBRT, and it could postpone the beginning of the ADT. 

At the same time, a greater experience in the treatment with VMAT-FFF could guarantee a valid alternative in prostate retreatments in those centers that do not have a Cyberknife^®^ system, or for those patients who are not compliant with long-term treatments or invasive procedures such as insertion of fiducial markers. It could allow in these cases to reduce the expected toxicities and give the basis to increase the dose to the target. Further prospective studies and a longer follow-up are needed to confirm the interest of reirradiation and to accurately identify the eligible patient population, appropriate for a second salvage radiation therapy. 

## Figures and Tables

**Table 1:** Characteristics of patients and of the first radical radiotherapy.

Gleason Score	No.	T Stage	No.
6(3 + 3)	10	1c	5
7(3 + 4)	4	2a	2
7(4 + 3)	5	2b	6
8(3 + 5)	1	2c	6
8(4 + 4)	5	3a	4
9(4 + 5)	1	3b	3
9(5 + 4)	2	4	1
Unknown	1	Unknown	2
**PSA at diagnosis (ng/mL)**	**No.**	**Age at diagnosis (ys)**	**No.**
<10			
10–20	13	50–60	7
>20	9	61–70	16
Mean (38.83)	7	71–80	6
Median (11)		>80	0
Range (3.53–345)			
**Radiotherapy (Total Normalized Dose)**	**No.**	**Radiotherapy (Fraction Dose)**	**No.**
64–84 Gy	25	1.8–2.0 Gy	25
35 Gy	3	5 Gy	3
38 Gy	1	9.5 Gy	1
**Technique of Radiotherapy**	**No.**	**Time between 1st RT and 1st relapse (ys)**	**No.**
3DCRT	18		6
IMRT	3	<4	13
VMAT	4	4–8	4
SBRT	3	9–10	6
BT	1	>10	

Abbreviations: GS = Gleason Score; T stage = primary tumor stage according to TNM staging system; 3DCRT = Radiotherapy 3D Conformal; IMRT = Intensity Modulated RadioTherapy; VMAT = Volumetric Modulated Arc Radiotherapy; SBRT = Stereotactic Body RadioTherapy; BT = brachytherapy; RT = radiation therapy.

**Table 2:** OARs and constraints.

OAR	Dose Limit	OAR	Dose Limit	OAR	Dose Limit
Bladder	V_30 Gy_ < 10% V_15 Gy_ < 40% D_30%_< 3.94 Gy	Rectum	V_30 Gy_ < 5% V_27 Gy_ < 10% V_24 Gy_ < 20% V_15 Gy_ < 50% V_21 Gy_ < 25 cc D_30%_ < 8.4 Gy D_60%_ < 4.08 Gy	Penile Bulb	V_29.5 Gy_ < 50% V_24 Gy_ < 50%

Abbreviations: OAR = organ at risk; Gy = gray; V = volume; D = dose.

**Table 3:** Patient characteristics and treatment.

Age at Recurrence (y)	No.	PSA at Re-RT (ng/mL)	No.	T-Restaging	No.	Restaging Imaging	No.
		<1	6	2a	4	MRI	1
50–60	0	1–2	9	2b	10	PET-CH	5
61–70	10	2.1–3	4	2c	4	PET-CH/MRI	9
71–80	15	3.1–4	4	3a	4	PET-CH/BS	1
>80	4	4.1–5	2	3b	3	PET-PSMA	2
		5.1–10	2	4	4	PET-PSMA/MRI	10
		>10	2			PET-FDG/MRI	1
**Time between 1st RT and re-RT (y)**	**No.**	**Re-RT technique**	**No.**	**Dose/n.fx**	**No.**	**ADT at first RT**	**No.**
						No	7
<4	5	Cyberknife^®^		30 Gy/5 fx	27	Yes	22
4	4	VMAT-Clarity^®^	17	35 Gy/5 fx	1	Duration (mo)	
5–10	16		12	30 Gy/3 fx	1	≤12	8
11–17	4					13–36	7
						>36	7

Abbreviations: RT = radiation therapy; ADT = androgen deprivation therapy; MRI = magnetic resonance imaging; PET = positron emission tomography; CH = coline; BS = bone scan; PSMA = prostate specific membrane antigen; FDG = fluorodeoxyglucose.

**Table 4:** Outcomes of patients.

	Follow-Up (Months)—Patients (pts)—Percentage (%)			
Patients at Risk at:	3 Months	6 Months	9 Months	12 Months
Freedom from Local Recurrence				
CK^®^ pts	16/17 (94%)	15/17 (88%)	14/16 (86%)	13/15 (87%)
VMAT-Clarity^®^ pts	11/12 (92%)	11/12 (92%)	11/12 (92%)	11/12 (92%)
Tot pts	27/29 (93%)	26/29 (90%)	25/28 (89%)	24/27 (89%)
Freedom from Distant Metastases				
CK^®^ pts	15/17 (88%)	15/17 (88%)	14/16 (86%)	13/15 (87%)
VMAT-Clarity^®^ pts	12/12 (100%)	12/12 (100%)	10/12 (83%)	10/12 (83%)
Tot pts	27/29 (93%)	27/29 (93%)	24/28 (86%)	23/27 (85%)
Freedom from Androgen Deprivation Therapy (ADT)				
CK^®^ pts	9/17 (53%)	9/17 (53%)	8/16 (50%)	8/15 (53%)
VMAT-Clarity^®^ pts	8/12 (67%)	8/12 (67%)	5/12 (42%)	5/12 (42%)
Tot pts	17/29 (59%)	17/29 (59%)	13/28 (46%)	13/27 (48%)
Freedom from Biochemical Relapse				
CK^®^ pts	16/17 (94%)	15/17 (88%)	14/16 (88%)	13/15 87%)
VMAT-Clarity^®^ pts	10/12 (83%)	10/12 (83%)	10/12 (83%)	8/12 (67%)
Tot pts	26/29 (90%)	25/29 (86%)	24/28 (86%)	22/27 (81%)

**Table 5:** Dosimetric data.

Prostatic Gland	GTV cm^3^	PTV cm^3^	D_max_ PTV	Rectum D_30%_ (<8.4 Gy)	Rectum D_60%_ (<4.08 Gy)	Bladder D_30%_ (<3.94 Gy)	Penis Bulb cm^3^	Penis Bulb (D_max_)	Penis Bulb V_29.5 Gy_ (<50%)	Penis Bulb V_24 Gy_ (<50%)	Bladder V_15 Gy_ (<40%)	Bladder V_30 Gy_ (<10%)	Rectum V_15 Gy_ (<50%)	Rectum V_21 Gy_ (<25 cc)	Rectum V_24 Gy_ (<20%)	Rectum V_27 Gy_ (<10%)	Rectum V_30 Gy_ (<5%)
CK 1	27.74	54.07	36.59	11.60	3.78	11.88	5.38	22.54	0.00	0.00	21.40	4.90	22.00	6.48	7.00	4.00	1.30
CK 2	34.40	69.68	36.59	12.62	7.23	16.38	15.85	31.47	1.10	8.70	34.10	6.90	22.10	5.85	5.30	2.40	0.40
CK 3	39.74	73.25	36.59	8.79	5.48	11.14	20.23	18.02	0.00	0.00	18.00	2.60	12.40	6.30	3.70	2.00	0.50
CK 4	65.52	119.63	38.46	16.17	8.32	14.40	5.00	28.63	0.00	12.30	27.80	4.20	33.80	15.5	11.70	7.20	3.70
CK 5	64.60	110.25	37.04	10.36	4.45	9.94	7.43	23.75	0.00	0.00	10.90	2.50	16.10	6.89	4.80	2.60	0.70
CK 6	40.24	77.00	38.96	13.77	8.16	18.73	8.12	30.34	0.90	14.10	42.50	7.40	25.90	7.05	9.80	6.00	2.20
CK 7	23.74	50.62	37.50	15.71	5.90	11.16	7.32	16.74	0.00	0.00	19.30	3.00	31.50	13.86	14.00	9.10	4.40
CK 8	39.72	78.60	37.50	10.71	6.84	14.00	11.83	33.26	5.10	14.50	27.70	8.00	17.10	6.32	6.20	4.10	2.10
CK 9	25.69	56.22	37.04	12.33	6.10	6.40	9.40	19.75	0.00	0.00	3.00	0.00	20.00	5.07	4.90	2.80	1.10
CK 10	22.67	47.86	37.50	11.85	6.91	9.63	7.24	29.08	0.00	11.90	15.70	3.70	20.20	5.35	6.50	3.80	1.30
CK 11	24.89	54.93	37.50	12.69	7.22	8.47	1.42	30.99	11.90	86.00	13.30	4.90	22.00	6.29	4.80	5.20	2.70
CK 12	50.31	90.12	37.50	8.80	3.34	6.83	13.01	28.38	0.00	6.70	11.00	1.90	15.10	7.03	5.00	2.40	0.50
CK 13	31.10	57.05	38.46	13.54	5.24	11.18	7.27	13.86	0.00	0.00	25.10	6.70	26.50	10.23	10.30	6.40	2.90
Median (range)	34.40 (22.67–65.52)	69.68 (47.86–119.63)	37.5 (36.59–38.96)	12.33 (8.79–16.17)	6.1 (3.34–8.32)	11.16 (6.4–18.73)	7.43 (1.42–20.23)	28.38 (13.86–33.26)	0 (0–11.9)	6.7 (0–86)	19.3 (3–42.5)	4.2 (0.1–8.0)	22 (12.4–33.8)	6.48 (5.07–15.5)	6.2 (3.7–14)	4 (2–9.1)	1.3 (0.4–4.4)
Clarity 1	81.78	136.33	30.19	18.03	13.07	16.17	8.20	30.26	22.85	70.84	33.27	1.84	46.87	12.03	13.1	7.98	0.00
Clarity 2	31.68	66.16	30.31	14.31	9.11	8.89	3.38	21.96	0.00	0.00	22.79	0.5	27.04	8	9.23	6.21	0.00
Clarity 3	52.39	95.22	30.25	14.89	10.34	9.42	1.98	30.12	7.45	31.44	16.43	1.29	29.79	9.67	10.69	6.2	0.64
Clarity 4	23.75	51.40	30.23	16.91	13.73	19.32	3.74	29.96	31.50	76.27	34.46	0.14	52.53	7.51	4.96	2.17	0.00
Clarity 5	50.51	95.31	30.21	18.12	13.36	26.93	4.50	29.9	14.68	44.6	70.27	5.07	46.11	10.44	15.49	10.26	0.00
Clarity 6	33.59	65.18	30.20	14.36	10.69	10.81	3.97	30.17	17.11	42.11	23.65	2.25	27.08	7.46	10.13	6.33	0.33
Clarity 7	27.71	60.38	30.25	17.15	9.20	17.55	2.78	30.02	23.86	63.61	38.13	2.15	37.64	12.49	14.19	8.89	0.84
Clarity 8	19.7	42.73	30.04	12.92	2.35	1.66	4.96	29.58	12.41	68.17	7	0	22.75	9.64	5.33	2.94	0.00
Clarity 9	25.44	51.72	30.16	19.46	8.36	9.57	6.39	29.40	0.59	43.46	23.41	3.24	45.24	16.79	15.95	9.07	0.23
Clarity 10	19.91	51.05	30.46	14.97	5.07	0.99	4.91	30.00	13.84	45.96	4.64	0.47	29.9	8.12	6.93	3.94	0.28
Median (range)	29.69 (19.7–81.78)	62.78 (42.73–136.33)	30.22 (30.04–30.46)	15.94 (12.92–19.46)	9.77 (2.35–13.73)	10.19 (0.99–23.93)	4.23 (1.98–8.2)	29.98 (21.96–30.26)	14.26 (0–31.5)	45.28 (0–76.27)	23.53 (4.64–70.27)	1.56 (0–5.07)	33.77 (22.75–52.53)	9.65 (7.46–16.79)	10.41 (4.96–15.95)	6.27 (2.17–10.26)	0.11 (0–0.84)
FFF-Clarity 1	81.78	136.33	37.94	6.72	3.82	13.13	8.20	34.76	25.41	47.89	26.18	4.43	8.54	2.56	2.55	1.21	0.23
FFF-Clarity 2	31.68	66.16	39.31	5.89	2.80	4.65	3.38	13.76	0.00	0.00	17.68	4.44	10.29	3.17	2.91	1.10	0.10
FFF-Clarity 3	52.39	95.22	37.02	5.45	2.55	3.62	1.98	31.18	1.45	15.51	13.14	3.43	13.49	5.43	6.47	4.25	2.01
FFF-Clarity 4	23.75	51.40	37.38	5.65	2.61	10.13	3.74	34.00	21.93	47.20	25.17	9.06	5.53	1.63	1.16	0.38	0.01
FFF-Clarity 5	50.51	95.31	37.37	6.29	3.83	26.24	4.50	32.87	11.61	28.66	67.01	13.95	10.70	2.76	3.52	1.66	0.25
FFF-Clarity 6	33.59	65.18	38.36	5.27	2.80	9.34	3.97	34.25	14.50	32.48	22.07	3.63	12.02	4.01	5.26	3.35	1.59
FFF-Clarity 7	27.71	60.38	39.16	6.26	2.52	21.44	2.78	31.75	18.46	53.49	50.11	6.32	12.91	4.79	5.29	3.03	1.05
FFF-Clarity 8	19.70	42.73	36.97	3.88	1.01	1.05	4.96	31.88	10.10	37.34	5.28	0.76	8.04	4.30	2.07	0.67	0.02
FFF-Clarity 9	25.44	51.72	39.21	5.87	1.65	4.26	6.39	28.68	0.00	10.33	16.09	5.85	12.18	4.80	4.82	2.63	0.63
FFF-Clarity 10	19.91	51.05	38.17	5.92	1.64	0.89	4.91	33.37	19.77	51.39	4.78	1.39	12.76	5.29	5.31	2.93	0.88
Median (range)	29.69 (19.7–81.78)	62.78 (42.73–136.33)	38.05 (36.97–39.31)	5.88 (3.88–6.72)	2.58 (1.01–3.83)	6.99 (0.89–26.24)	4.23 (1.98–8.2)	32.37 (13.76–34.76)	13.05 (0–25.41)	34.91 (0–53.49)	19.87 (4.78–67.01)	4.43 (0.76–13.95)	11.36 (5.53–13.49)	4.15 (1.63–5.43)	4.17 (1.16–6.47)	2.14 (0.38–4.25)	0.44 (0.01–2.01)

**Table 6:** Literature search: type of radiotherapy, dose, fractionation, follow-up, target, and androgenic deprivation therapy.

Author Year (n.pt)	Age Year (Range)	Type of Study	First Treatment Modality	Relaps Time Months (Median)	FU Median Months (Range)	PSA Pre-SBRT Median (Range)	SBRT	RT (Total Dose/n.f) (Daily Fx)	Target	ADT at Reirradiation
Caroli et al. [15], 2020 (p. 38)	75 (71–80)	R	RT (11) RP + RT (27)	-	27 (4–35)	1.10 (0.82–2.59)	Tomotherapy	18 Gy/3 (6)	Prostatic bed	-
Fuller et al. [16], 2019 (p. 50)	74 (50–89)	P	EBRT (3DCRT-IMRT-PT)	98 (32–241)	44 (3–110)	3.97 (0.1–48.6)	CK	34 Gy/5 (6.8)	Prostate gland	7(+)
Fuller et al. [10], 2015 (p. 29)	73 (50–89)	P	EBRT (27) BT (1) SBRT (1)	88 (32–200)	24 (3–60)	3.1 (0.1–48.2)	CK	34 Gy/5 (6.8)	Prostate gland	7(+)
Jereczek-Fossa et al. [17], 2012 (p. 15)	68.3 (57–82)	R	EBRT (88%) BT (3%)	66 (24–180)	9.5 (3–28.9)	3.51 (1.69–22.9)	CK	30 Gy/5 (6)	Prostate gland	5(+)
Loi et al. [9], 2018 (p. 50) Francolini et al. [18], 2022 (long-term results)	76 (62–86)	R	EBRT (28) RP (22)	76 (9–205)	48.2 (6.4–86.3)	10 (3.1–160)	CK	30 Gy/5 (6)	Dominant intraprostatic lesion Prostate bed	11(+)
Janoray et al. [19], 2016 (p. 21)	73 (59–85) 80 (91–68)	R	EBRT (11) RP (10)	98.4 (37.9–246) 98.03 (43.4–398)	11.7 (2.5–46.5)	3.43 (1.65–24.1) 3 (0.42–14.5)	CK	3 pz 35 Gy/5 (7) 15 pz 36.25 Gy/5 (7.25) 3 pz 36 Gy/6 (6)	Prostate gland Locoregional recurrence	1(+) 1(+)
Mbeutcha et al. [20], 2017 (p. 28)	69 (65–77) 69 (64–75)	R	BT-HDR (16) EBRT (12)	69 (55–85) 49 (37–70)	22.5 (8–42) 14.5 (7–23)	4.37 (2.01–4.76) 4.5 (3.0–6.3)	BRT CK	35 Gy/5 (7)	Prostate gland	2(+) 10(+)
Zerini et al. [21], 2015 (p. 32)	73 (60–83)	R	EBRT 29 BT 3	99.7 (23–208.4)	21.3 (2–53)	3.9 (0.8–16.9) 2.3 (0.7–51.8)	VMAT	25–30 Gy/5–10 (3–6) 15–25 Gy/3–5 (5)	Prostate gland 22 Prostate bed 10	8(+) 3(+)
Leroy et al. [22], 2017 (p. 23)	-	R	EBRT 19 (83%) BT 4 (17%)	65 (28–150)	22 (6–40)	2.5 (0–11.7)	CK	36 Gy/6 (6)	Whole prostate 19 Hemi-prostate 1 Focal treatment 3	14(+)
Miszczyk et al. [23], 2018 (p. 38)	71.6 (59–89)	R	RP + EBRT (3) EBRT (31) RP + EBRT + BT (1) EBRT + BT (2) BT (1)	101 (22–179)	14.4 (1.6–46.4)	3.26 (0.12–48.83)	CK	36.25 Gy/5 (7.25) 36 Gy/6 (6); 30 Gy/5 (6) 30 Gy/2 (10) + 10 boost 30 Gy/3 (10); 18 Gy/3 (6) 20 Gy/2 (10); 22.5 Gy/3 (7.5) 27.5 Gy/5 (5.5)	Prostate gland 1 lobe Local relapse	21(+)
Detti et al. [24], 2015 (p. 16)	65 (52–78)	R	RP + EBRT (8) RP (8)	126 (42–256)	10 (2–21)	4.1 (0.5–11.09)	CK	30 Gy/5 (8) 35 Gy/5 (8)	Prostatic bed	0(+)
Bergamin et al. [25], 2020 (p. 25)	72 (62–83)	R	EBRT (21) EBRT + BT HDR (2) BT LDR (2)	99 (54–163)	25 (16–46)	4.1 (1.1–16.6)	IMRT/VMAT	36 Gy/6 (6) 38 Gy/6 (6.3)	Prostate gland	0(+)
D’Agostino et al. [26], 2019 (p. 23)	78 (69–85)	R	RP + RT (8) RT (15)	90 (26–138)	33(6–58)	3.2 (1.2–13.5)	VMAT	30 Gy/5 (6)25 Gy/5 (5)	Prostate gland (13) Prostatic bed (8) Prostate and local recurrence (2)	8(+)
Jereczek-Fossa et al. [27], 2019 (p. 64)	73.2 (52.6–81.7)	R	EBRT (59) EBRT + BT (1) BRT-LDR (4)	99.7 (23–208.4)	26.1 (3.1–82.4)	3.89 (0.17–51.8)	IMRT(VERO) (54) IMRT(Trilogy) (7) CK (3)	30 Gy (20–30)/5 (2–10)	Prostate gland (40) PPI (4) Prostate gland + boost (1) Prostatic surgical bed (19)	16(+)
Ozyigit et al. [28], 2020 (p. 11)	71 (59–86)	R	EBRT	63 (23–178)	19	2.33	CK VMAT (Novalis Versa-HD)	30 Gy/5 (6)	Focal reirradiation	-
Lewin et al. [29], 2021 (p. 30)	62 (52–75)	R	EBRT (25) BT (5)	72 (18–176)	28	3.63 (0.05–77)	VMAT	32.5 Gy/5 (6.5)	Prostate gland (18) Prostate + SV (10) SV (2)	11(+)
Vavassori et al. [30], 2010 (p. 6)	68 (63–74)	R	EBRT (6)	13.5 (2.7–38.4)	11.3 (9.6–18.6)	3.65 (2.1–14.1)	CK	30 Gy/5 (6)	Prostate gland (6)	5(+)
Oliver et al. [31], 2019 (p. 12)	58	R	RP + EBRT	6.5 (1–116)	77.6 (21.4–160.8)	1.13 (0.57–5.71)	CK	36 Gy/6 (6)	Prostatic bed (12)	2(+)
Our study, 2022 (p. 29)	73 (61–86)	R	EBRT (25) SBRT (3) BT (1)	72 (12–204)	27 (6–60)	1.9 (0.2–17)	CK (17) VMAT-IGRT-Clarity (12)	30 Gy/5 (6)	Prostate gland (23) Prostatic bed (4) Intraprostatic lesion (2)	12(+)

**Table 7:** Literature search: toxicity, local control/failure, and constraints.

Author, Year (n. pts)	Toxicity	Toxicity (Criteria)	Local Control/Failure	Constraints
Caroli et al. [15], 2020 (p. 38)	Acute GU G1: 31.6%	CTCAE v.4.0	15mo 95%	-
Fuller et al. [16], 2019 (p. 50)	Acute GU G1–G2: 2% Late GU G2 17%; G3 8%	CTCAE v.3.0	2y 76%; 5y 60% LRF 94%; DRF 89%	Urethra: D_max_ < 120%; D_50_ < 105%; Rectum: D_max_ < 75%; Rectal wall; D_max_ < 100% Bladder wall: D_max_ < 100%
Fuller et al. [10], 2015 (p. 29)	Acute GU G3: 3% Late GU G2: 10%; G3: 3%; G4: 3%	CTCAE v.3.0	BDFS 2y 82% CDFS 2y 100%	Urethra: D_max_ < 120%; D_50_ < 105% Bladder wall: D_max_ < 100%; Rectal wall; D_max_ < 100%
Jereczek-Fossa et al. [17], 2012 (p. 15)	Acute GU: G1 2: 13%; G2: 13%; G3: 7% Late GU: G1 7%; G2 7%; G3 7%	RTOG	PFS 30mo: 42.6% DP: 5pz	Bladder: D_max_ < 120%; Rectum: D_max_ < 100%; Small bowel: V_21 Gy_ < 1 cc
Loi et al. [9], 2018 (p. 50) Francolini et al. [18], 2022 (long-term results)	Acute GU: G1 18%; G2 2%; G3 2%; Acute GI: G1 8% Late GU: G1 18%; G2 6%; G3 2%; Late GI: G1 2%; G2 4%; G3 2%	CTCAE v.4.03	BRFS 1y 80%; DMFS 1y 92% BRFS 2y 50%; DMFS 2y 82%	Bladder:D_max_ < 120%; Rectum: D_max_ < 100% Small bowel V_21 Gy_ < 1 cc
Janoray et al. [19], 2016 (p. 21)	Acute: GU: G1 14%; G2 5% Acute GI: G1 10% Late: GU: G1 5%	CTCAE v.4.0 IPSS	BRFS 1y 83.3–85.7% Local Failure 1y 4%	Rectum: V_18.1 Gy_ < 50%; V_29 Gy_ < 20%; V_36 Gy_ < 1 cc; Bladder: V_18.1 Gy_ < 40%; V_37 Gy_ < 10 cc Femoral head: V_14.5 Gy_ < 5%
Mbeutcha et al. [20], 2017 (p. 28)	BT Acute GU: G1 30%; G2 40%; Late GU: G1 50%; G2 10% CK Acute GU: G1 27.8%; G2 11.1%; Acute GI: G1 5.6%; G2 11.1% CK Late GU: G1 22%; G2 5.6%; CK Late GI: G2 5.6%	CTCAE v.4.03	BT: BFFS 44.4% CK: BFFS 44.4%; 33.3%	Urethra: V_115_ < 1% Rectum: V_80_ < 1%
Zerini et al. [21], 2015 (p. 32)	Prostate Acute: GU: G1 14%; G2 6%; GI: G1 9%; Late: GU: G1 19%; GI: G1 12% Prostatic bed Acute: GU: G1 3%; GI: G2 3%; Late: GU: G2 3%; GU: G1 3%	RTOG/EORTC	BF 1y 9% CF 1y 37% DFS 2y 40.6% OS (21.3mo): 93.7%	Prostate reirradiation Rectum: D_30%_ < 13.8 Gy; D_60%_ < 6.69 Gy; Bladder: D_30%_ < 10.58 Gy Prostatic bed irradiation Rectum: D_30%_ < 8.4 Gy; D_60%_ < 4.08 Gy; Bladder: D_30%_ < 3.94 Gy
Leroy et al. [22], 2017 (p. 23)	Acute GU: G1 47%; G2 30%; G3 9% Acute GI: G1 8.7%; G2 8.7%	CTCAE v.4.0	2y DFS 54%; OS 100%	Rectum: V_27 Gy_ < 2 cc; V_12 Gy_ < 20%; Bladder: V_27 Gy_ < 5 cc; V_12 Gy_ < 15% Intra-prostatic urethra: V_24 Gy_ < 30%; V_36 Gy_ < 1 cc
Miszczyk et al. [23], 2018 (p. 38)	Acute GU: G1 31.8%; G2 13%; G3 3.7%; Acute GI: G1 7.4% Late GU: G1 22.2%; G2 16.7%; G3 12.5%; Late GI G1 11.1%; G2 4.8%	RTOG/EORTC	BF: 13.2% BFFS 86.8%	-
Detti et al. [24], 2015 (p. 16)	Acute GU: G2 6% Acute GI: G2 6%	CTCAE v.4.0	BRR 88%	Rectum: D_30%_ < 18.8 Gy; D_60%_ < 10 Gy; Bladder: D_40%_ < 18.1 Gy; D _50%_ < 16.6 Gy Urethra: D_max_ < 33.7 Gy; Dmean < 31 Gy; Femoral heads: V_14.5 Gy_ < 5%; Penile bulb: V_29.5 Gy_ < 50%
Bergamin et al. [25], 2020 (p. 25)	Acute GU: G1 24%; G2 4% Acute GI: G1 8%; G3 4% Late GU: G1 28%; G2 4% GI: G1 8%	CTCAE v.4.03 RTOG/EORTC	BFFF 2y 80% 5y 35–82%	Rectum: D_0.1 cc_ < 33 Gy; D_0.5 cc_ ≤ 28 Gy; D_1.0 cc_ < 24 Gy D_2.0 cc_ ≤ 18 Gy Bladder: D_0.1 cc_ ≤ 33 Gy; D _0.5 cc_ ≤ 28 Gy; D _1.0 cc_ ≤ 24 Gy; D _2.0 cc_ ≤ 18 Gy Urethra: D_max_ < 33 Gy; Urethra PRV: D_max_ < 36 Gy
D’Agostino et al. [26], 2019 (p. 23)	Acute GU: G1 43%; G2 13%; G3 4% Late GU: G1 17%; G3 4%	CTCAE v.4.03	BRFS 1y 81.6%; 2y 41.7% LC 1y 95%; 2y 61.1% PFS 1y 85.9%; 2y 63.6%	Rectum: V_10 Gy_ < 40%; V_18 Gy_ < 20% Bladder: V_10 Gy_ < 25%; V_18 Gy_ < 15% Femoral heads: V_24 Gy_ < 10%; Penile bulb: V_24 Gy_ < 50% Small intestine: V_18 Gy_ < 5 cm^3^
Jereczek-Fossa et al. [27], 2019 (p. 64)	Acute GU: G1 20%; G2 5%; G3 1.5%; Acute GI: G1 8%; G2 2% Late GU: G1 28%; G2 9%; G3 1.5%; Late GI: G1 6%; G2 1.5%	RTOG/EORTC	2y LC: 75%; BFFS: 40%; CFS: 53%	Rectum: D_30%_ < 13.5 Gy; D_60%_ < 6.7 Gy Bladder: D_30%_ < 10.6 Gy
Ozyigit et al. [28], 2020 (p. 11)	Acute GI: G3 9% Late GU: G2 9%	CTCAE v.4.0 RTOG/EORTC	BFFS 1y 89%; 2y 48%	Bladder: D_max_ < 120%; Rectum: D_max_ < 100%; Small bowel: V_21 Gy_ < 1 cc
Lewin et al. [29], 2021 (p. 30)	Acute GU: G2 27%; G3 3% Late GU: G2 30%; G3: 3%; Late GI: G2 3%	CTCAE v.4.0	BF 2y 53%; CF 33% RFS 2y 60%; RFS 3y 53%	Rectal wall V_100%_ < 5%; V_90%_ < 15%; V_80%_ < 20%; V_38 Gy_ < 2 cc
Vavassori et al. [30], 2010 (p. 6)	Acute GI: G1 33%; Late GU: G1 33%	RTOG/EORTC	BF 1y 66%; CF 1y 50%	Urethra D_max_ < 125%; Rectum D_max_ < 75%
Oliver et al. [31], 2019 (p. 12)	Acute GU: G1–2 25%; Acute GI: G1–2 8% Late GU: G1-2 12.5%	CTCAE v.4.0	BRFS 1y 79%; BRFS 2y 56%	Rectum V_12 Gy_ < 20%; V_27 Gy_ < 2 cc; Bladder V_12 Gy_ < 15%; V_27 Gy_ < 5 cc
Our study, 2022 (p. 29)	CK Acute GU: G2 23%; Acute GI: G2 6% VMAT-Clarity Acute GU: G2 17%; VMAT-Clarity Late GU: G2 8%	RTOG/EORTC	LRF 1y 89%; DRF 1y 85% BRFS 2y 81%	Rectum: V_30 Gy_ < 5%; V_27 Gy_ < 10%; V_24 Gy_ < 20%; V_15 Gy_ < 50%; V_21 Gy_ < 25 cc; D_30%_ < 8.4 Gy; D_60%_< 4.08 Gy Bladder: V_15 Gy_ < 40%; V_30 Gy_ < 10%; D_30%_ < 3.94 Gy; Penis bulb V_29.5 Gy_ < 50%; V_24 Gy_ < 50%

## Data Availability

The data will be available as raw data to URL: Reirradiation of locally recurrent prostate cancer with Cyber-knife^®^ System or Volumetric Modulated Arc Therapy (VMAT) and IGRT-Clarity^®^: outcomes, toxicities and dosimetric evaluation—Zenodo (https://zenodo.org/record/6580660#.Yo4wq-7P23A, accessed on 15 May 2022).
